# The asymmetry engine: how plants harness asymmetries to shape their bodies

**DOI:** 10.1111/nph.20413

**Published:** 2025-01-28

**Authors:** Kristoffer Jonsson, Anne‐Lise Routier‐Kierzkowska, Rishikesh P. Bhalerao

**Affiliations:** ^1^ Department of Biological Sciences, IRBV University of Montreal 4101 Sherbrooke Est Montreal QC H1X 2B2 Canada; ^2^ Department of Forest Genetics and Plant Physiology, Umeå Plant Science Centre (UPSC) Swedish University of Agricultural Sciences 901 83 Umeå Sweden

**Keywords:** auxin, cell wall, development, feedback mechanisms, growth coordination, mechanics, morphogenesis, tropism

## Abstract

Plant development depends on growth asymmetry to establish body plans and adapt to environmental stimuli. We explore how plants initiate, propagate, and regulate organ‐wide growth asymmetries. External cues, such as light and gravity, and internal signals, including stochastic cellular growth variability, drive these asymmetries. The plant hormone auxin orchestrates growth asymmetry through its distribution and transport. Mechanochemical feedback loops, exemplified by apical hook formation, further amplify growth asymmetries, illustrating the dynamic interplay between biochemical signals and physical forces. Growth asymmetry itself can serve as a continuous cue, influencing subsequent growth decisions. By examining specific cellular programs and their responses to asymmetric cues, we propose that the decision to either amplify or dampen these asymmetries is key to shaping plant organs.

**Table:** 

	Contents	
	Summary	2422
I.	Introduction	2422
II.	Externally guided growth asymmetry	2423
III.	Internally derived growth asymmetry	2423
IV.	Cues arising as a result of bending	2424
V.	Why do some organs bend and others grow straight?	2424
VI.	Conclusions	2426
	Acknowledgements	2426
	References	2426

## I. Introduction

Growth asymmetry is fundamental for the proper development of plants, influencing how they establish body plans and respond to environmental signals throughout their life cycle. While growth asymmetry can occur at various scales – from subcellular processes to organ‐level dynamics – this review focuses on how asymmetry is initiated, propagated, and regulated through feedback mechanisms, particularly those involving mechanochemical cues and hormonal signals.

We propose that growth asymmetry functions not only as a response to external and internal signals but also as a morphogenetic driver that shapes plant form and structure. To conceptualize this, we introduce the idea of an *asymmetry engine* – a biological mechanism by which plants harness spatially and temporally asymmetric signals, such as auxin gradients and mechanical stresses, to fuel coordinated growth and morphogenesis. Much like a mechanical engine transforms energy into motion, this process translates differential biochemical and mechanical cues into organized growth patterns, guiding organ shaping and enabling plants to adapt to fluctuating environmental conditions. Through a continuous cycle of dynamic feedback, plants navigate the decision to either amplify or counteract asymmetric cues, which dictates the direction and magnitude of growth and shapes their organs.

By integrating external and internal cues, plants coordinate and fine‐tune their asymmetric growth patterns through dynamic feedback loops. This coordination ensures the precise execution of morphogenetic processes and robust organ shaping, even under fluctuating environmental conditions. The ongoing regulation of these asymmetries not only enables plants to adapt to changing environments but also supports overall developmental flexibility and architectural robustness.

## II. Externally guided growth asymmetry

Asymmetric growth patterns may arise in response to internal or external cues. By receiving an asymmetric external signal, such as unidirectional light, growth promoters such as plant hormones orchestrate growth asymmetry. The role of plant growth regulator indole‐acetic acid (IAA/auxin) is particularly well‐studied in the regulation of differential growth (Retzer *et al*., 2014). Auxin is actively shuttled, via PINs and other auxin transporters, preferentially to one side of the responding organ. The resultant hormonal concentration gradient induces growth asymmetry, which leads to organ deformation (e.g. bending or curving) and reorientation. Gravisensitive organs, such as roots and shoots, respond to inclinations deviating from the gravity vector by establishing hormonally induced growth asymmetries to realign themselves accordingly. Interestingly, the gravity responses of young roots and hypocotyls both involve a statolith sedimentation‐guided activation of mechanosensitive ion channels that alters auxin transport toward the lower side of the organ (Baldwin *et al*., 2013). However, while increased auxin causes root cells to decelerate their growth, hypocotyl cells respond by accelerating expansion, thus producing opposing behaviors (Fendrych *et al*., 2016; Barbez *et al*., 2017), likely underpinned by organ‐specific differences in auxin response machineries and networks of hormonal cross‐talk. As a relay of the asymmetric signal, auxin induces local cell wall modifications that facilitate differential cell expansion across the organ (Barbez *et al*., 2017; Jonsson *et al*., 2021). External signals are thus fundamental in guiding growth asymmetry and orchestrating the processes that shape plant architecture. These signals provide key instructions for how plants respond to their environment, influencing overall morphogenesis and organ development.

In the following sections, we outline how external signals interact with intrinsic cues, and together, how their coordination drives the fine‐tuning of morphogenesis, ensuring adaptive and precise growth.

## III. Internally derived growth asymmetry

While external cues such as light, gravity, and temperature are key drivers of adaptive growth behaviors, endogenous signals provide a robust framework that ensures the coordination and consistency of morphogenetic events. Plants grown in the absence of light or in microgravity display overall normal organ shapes, implying that morphogenesis relies on robust internal developmental programs. Even when the external signal appears dominant, different internal programs may fine‐tune the precise organ response. For example, primary roots, orienting their growth along the gravity vector, employ an arsenal of auxin transporters different from that of lateral roots, suggested to produce a stronger gravity response (Rosquette *et al.*, 2013). Lateral roots, which instead grow at a semi‐vertical angle, additionally employ cytokinin signaling on the upper side of the organ, locally dampening growth to counteract the growth asymmetry achieved by auxin‐induced growth repression on the lower side (Waidmann *et al.*, 2019). Interestingly, uniform external signals can induce asymmetric growth, as seen in leaf hyponasty, where petioles bend upward in response to flooding or shading, allowing leaves to rise above water or capture more light. This response presumably exploits pre‐existing structural asymmetries in petiole cell walls, with the abaxial side growing faster than the adaxial side (Rauf *et al*., 2013). Ethylene and auxin signaling drive this differential growth, with ethylene promoting auxin transport to the abaxial side and establishing a growth‐inducing gradient (Sandalio *et al*., 2016). However, the mechanisms polarizing auxin transport remain unclear and may involve inherent adaxial/abaxial differences in transport machinery or mechanical properties. While the growth asymmetry is sufficient to initiate bending, it is tempting to speculate that feedback mechanisms, such as changes in cell wall properties or auxin redistribution, could amplify the initial asymmetry, ensuring robust and sustained bending under fluctuating environmental conditions. The concept of asymmetry amplification is perhaps most striking during apical hook development, where a curved region of the hypocotyl shields cotyledons and the meristem during soil penetration (Mazzella *et al*., 2014). According to the current model, the hook forms via growth asymmetry, as the hypocotyl folds over one side that grows slower than the opposite side, due to its higher levels of auxin (Schwark & Schierle, 1992), similar to gravitropic response in roots (Retzer *et al*., 2014). In agreement with this model, transport‐mediated auxin asymmetry is essential for hook formation (Vandenbussche *et al*., 2010; Zádníková *et al*., 2010). However, the initiation of growth asymmetry appears to precede the establishment of auxin response asymmetry (Peng *et al*., 2022). While it is currently unclear whether other key regulators of hook development, such as ethylene, gibberellins, and brassinosteroids may mediate this initial asymmetry, a proposed feedback mechanism between wall mechanochemical properties and the auxin transport machinery could reconcile these findings. While the initial bending would be caused by an auxin‐independent growth asymmetry, changes in hypocotyl curvature would trigger directional auxin redistribution toward the inner side. High auxin levels on the inner side of the hook promote pectin methylesterification, locally stiffening the cell wall. We proposed that such a change in mechanical properties could positively feed back to the auxin machinery to further amplify the asymmetric auxin response (Jonsson *et al*., 2021). Other wall components, such as cellulose and xyloglucans, could also be implicated (Aryal *et al*., 2020; Baral *et al*., 2021) in this loop. Such mechanism for self‐amplifying growth asymmetry would result in a highly curved organ shape, breaking the original organ symmetry. This feedback loop plausibly involves some mechanical signaling, as hook bending and/or auxin asymmetry can be reconstituted by mechanical constraint in otherwise hook‐defective mutants such as *ktn1* (Baral *et al*., 2021). Initiation of hook bending by consolidating growth fluctuations is reminiscent of the proposed mechanism underlying organ outgrowth initiation in the apical meristem, whereby local growth fluctuations influence PIN‐dependent auxin flow toward local growth maxima, reinforcing the growth variations (Heisler *et al*., 2010; Uyttewaal *et al*., 2012). Thus, through specific cellular responses to these growth fluctuations, morphogenesis can be achieved by harnessing stochastic asymmetries, with internal signals serving as central instructions for guiding the bending and shaping of plant organs.

## IV. Cues arising as a result of bending

Changes in organ shape could serve as a dynamic cue and induce responses that instruct subsequent growth decisions. In straight growing stems such as hypocotyls, the outer tissue layer (the epidermis) is under axial tensile stress stemming from the inner layers, themselves compressed by the epidermis (Kutschera & Niklas, 2007). Such baseline patterns of mechanical stress are presumably crucial in guiding both amplitude and direction of growth (Baskin & Jensen, 2013). Whether arising via growth asymmetry or by external force, bending introduces mechanical stress asymmetry across the curving organ (Kutschera & Niklas, 2007; Jonsson *et al*., 2023), which has been proposed to be perceived by the plants through the resulting asymmetric strain (i.e. relative change in length) (Coutand, 2000; Coutand *et al*., 2009). For example, increased strain imposed by stem bending is accompanied by a corresponding rise in expression of the mechanosensitive gene *PtaZFP2*, suggesting a mechanism that can quantitatively sense the amount of bending experienced. Indeed, accurate modeling of the bending kinetics of oat coleoptile gravitropic responses required the inclusion of a curvature‐proportional proprioceptive component (Bastien *et al*., 2014). This hypothetical response of unknown molecular origin operates by counteracting the bending relative to the amplitude of curvature locally at each point along the organ. While this indicates that precise curvature sensing is at work, it does not offer any cell‐level mechanism. Numerous molecular mechanisms are activated upon bending, such as auxin transport (Ditengou *et al*., 2008; Kircher & Schopfer, 2016), FERONIA‐dependent Ca^2+^ signaling (Shih *et al*., 2014), and induction of mechanoresponsive (TCH) genes (i.e. activated in response to mechanical stimuli) (Tixier *et al*., 2014), demonstrating that plants possess a diverse array of potential pathways for sensing bending as a cue. Cortical microtubules (CMTs) have been proposed as possible mechanosensors (i.e. structures that detect mechanical forces). CMTs, which inform the direction of cellulose deposition in the cell wall, are highly responsive to different kinds of environmental cues, including mechanical forces, and realign in response to gravity‐induced growth asymmetry. Such MT realignment seems a result of the actual bending and not the gravity response (Ikushima & Shimmen, 2005): externally imposed bending causes the same MT effect. With both externally imposed and growth‐derived bending, MTs on the outer side of bent bean epicotyls become preferentially transversely oriented, while on the inner side of the bend they orient preferentially longitudinally. This outcome is not obvious when one considers that mechanical stress patterns arising from externally imposed bending vs growth asymmetry‐driven curving are opposing (see Jonsson *et al*., 2023). However, these findings can be reconciled if MTs act as strain‐sensors (detecting deformation caused by bending) rather than stress sensors (detecting force per unit area) as has been suggested (Moulia *et al*., 2021). Yet, further complicating the picture are observations during *Arabidopsis* apical hook bending, where cells on the outer side exhibit distinctly longitudinal orientation, while significantly less biased on the inner side (Baral *et al*., 2021), suggesting that we lack a complete understanding of mechanical stress landscapes in bending organs as well as CMT behavior in such systems. Other components may preferentially exhibit stress responsiveness during organ bending. Receptor‐like kinases, such as THESEUS and FERONIA (FER), have extracellular malectin‐binding domains that interact with cell wall components and are essential for mechanical stress responses (Hématy *et al*., 2007; Feng *et al*., 2018). Upon externally imposed root bending, an FER‐dependent pH increase occurs on the outer, stretched, side of the curve (Shih *et al*., 2014; Feng *et al*., 2018). FER is also necessary for alkalinization on the inner side during root gravitropic bending (Barbez *et al*., 2017). This apparent contradiction can be explained by different stress patterns: external bending causes tension on the outer side, while gravitropic bending, driven by growth asymmetry, can create tension on the inner side (Jonsson *et al*., 2023). Moreover, FER has been linked with PIN2 polarity, and mutations in PIN2/AUX1 can alleviate root nutating defects of *fer* (Dong *et al*., 2019; Li *et al*., 2020). Given FER's strong implication in mechanosensing, its connection to polar auxin transport – key for bending responses – suggests a compelling integration of mechanical input transduction and auxin transport machinery. This interplay provides a framework through which FER (and possibly other mechanosensors such as THESEUS) integrates mechanical cues, regulates auxin distribution, and generates feedback loops to coordinate bending responses. Together with the diverse bending‐responsive pathways described earlier, this connection underscores the intricate, multilayered mechanisms plants use to perceive and respond to bending stimuli, ensuring robust and adaptive growth.

## V. Why do some organs bend and others grow straight?

**Fig. 1:**
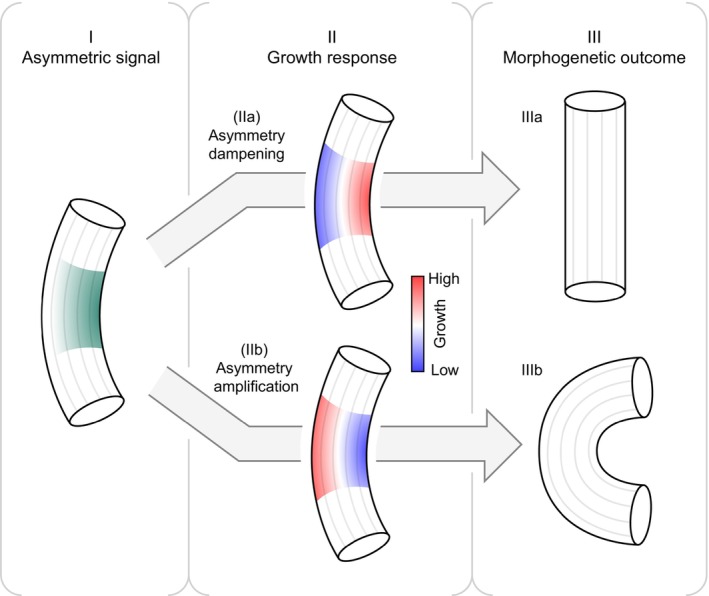
The asymmetry engine: a regulatory framework for plant morphogenesis. The *asymmetry engine* represents a dynamic regulatory framework that guides plant organ development by responding to asymmetric cues, whether from external signals (e.g. gravity, light) or internal instructions. At each stage of growth, plants face a fundamental decision: to amplify or dampen the asymmetry encountered. This framework orchestrates morphogenesis by dynamically adjusting growth patterns based on these cues. (I) Signal establishment: upon bending, an asymmetric signal gradient is generated across the organ, depicted here as a curvature accompanied by an inner‐to‐outer gradient. This gradient serves as the initial input. (II) Growth response: the asymmetric signal triggers localized growth responses, which can diverge into one of two pathways. In (IIa), the response dampens asymmetry by promoting faster growth on the inner side compared to the outer side. Conversely, in (IIb), the response amplifies asymmetry by favoring faster growth on the outer side relative to the inner side. (III) Morphogenetic outcome: the growth responses culminate in distinct structural outcomes. In (IIIa), the dampening response from (IIa) straightens the organ, yielding a flat shape. In (IIIb), the amplifying response from (IIb) accentuates the bend, resulting in a curved structure. Through this process, asymmetric cues and their local responses act as a unified guiding blueprint, ensuring that plants continuously adjust their growth to either reinforce or counteract asymmetry. This iterative decision‐making shapes the plant's form and adaptation throughout its life.

Bending itself may serve as a crucial cue for morphogenetic processes, but neither straight growth nor bending are universally adopted default growth modes. In the absence of external cues, some organs, such as stems and roots, exhibit largely straight and symmetric elongation, while others, such as tendrils and the apical hook, are primed for asymmetric growth. We suggest the difference might lie in the response to asymmetric cues. Stochastic fluctuation in growth is inherent to all biological systems, and cells experience a continuous stream of asymmetric growth cues (Moulia *et al*., 2021), which could locally elicit an acceleration or repression of growth, that either amplifies or dampens the initial asymmetry (Fig. 1). This is illustrated in the *Arabidopsis* hypocotyl, which achieves straight growth independently of gravity sensing (Tsuda *et al*., 2011; Gupta *et al*., 2012; Miyamoto *et al*., 2014) The CMT‐associated protein NEK6 has been proposed to buffer growth fluctuations by selectively depolymerizing longitudinal MTs. (Takatani *et al*., 2020). The nek6 mutant exhibits a hyperaligned longitudinal CMT network and a wavy growth pattern, suggesting an exaggerated growth compensation in response to minute stochastically derived growth asymmetries (Takatani *et al*., 2020), which may be perceived as oscillating waves of strain and/or compression/tension (Robinson & Kuhlemeier, 2018). A dampening mechanism has also been proposed for maintaining sepal flatness, which could rely on an auxin‐dependent machinery for fine‐tuning the growth balance between adaxial and abaxial sides by modulating cell wall properties in response to local cues (Xu *et al*., 2023).

In contrast to asymmetry prevention, the development of the apical hook seems to rely on internally derived stochastic growth variations for further amplification of growth asymmetry. Feedback mechanisms involving mechanochemical cues and auxin appear to drive this behavior (Jonsson *et al*., 2021), but the actual readout of asymmetry is likely a local phenomenon occurring at the cellular level. As a consequence of asymmetric organ growth, cells across a curving organ are exposed to different cues (Jonsson *et al*., 2023). This raises the question of what is different between tissues that counteract growth asymmetry and those which amplify it – the cue or the response? When comparing the apical hook and hypocotyl, very little is known about the difference in the underlying nature of growth asymmetry as a cue. We do, however, know more about the response. In both cases, auxin distribution becomes asymmetric during bending (Schwark & Schierle, 1992; Rakusová *et al*., 2011), but its downstream effects are opposite. In the hook, auxin is thought to inhibit cell expansion via wall modifications and alkalinization (Jonsson *et al*., 2021; Du *et al*., 2022), while, in bending hypocotyls, a relative increase in auxin promotes wall acidification and growth (Rakusová *et al*., 2011; Fendrych *et al*., 2016). It is thus conceivable that tissue‐specific transcriptional programs endow cells with a particular set of components such as proton pumps to respond locally to a cue such as mechanical tension and/or compression by either acidifying or alkalinizing the cell wall, and growing either more or less. Through this cell‐specific response, various tissues would be primed to either intensify or diminish underlying asymmetries (Fig. 1). However, the response program is probably highly transient, as cells that once resided in the apical hook subsequently migrate to the hypocotyl below, now occupying a tissue with an opposing response to asymmetry. How plants set up their local asymmetry response programs remains a mystery, and uncovering this will further our understanding of how plants are capable of generating such a diverse set of organs from a minimal set of cues.

## VI. Conclusions

It is evident from above that growth asymmetry is not just a response of the plants to external and internal signals but can also be a driving force in morphogenesis. The dynamic roles of auxin distribution and the feedback loops involving mechanochemical cues in bending highlight how plants shape their bodies and adapt to their environments via growth asymmetry. However, it is equally true that bending can feedback and, through similar components, influence the course of morphogenesis. For example, bending roots can initiate lateral roots across the bend. Understanding these processes at a fundamental level opens new avenues for deciphering the complexity of plant morphology and development. Future research will likely reveal more about the specific cellular programs that dictate whether asymmetries are amplified or counteracted.

A major open question in understanding the mechanistic basis of growth asymmetry remains the role of CMTs. CMTs have been proposed as mechanosensors since their very discovery (Green, 1962). Extensive studies such as in bending roots and young stems, led to a general model for organ bending, in which a tropic response triggers a switch in CMT orientation, resulting in asymmetric cellulose orientation and, ultimately, differential cell elongation (Blancaflor, 2002; Bisgrove, 2008). However, this model has been challenged by reports of normal tropic bending after de‐polymerizing or stabilizing CMTs (Nick *et al*., 1991; Hasenstein *et al*., 1999). Also, strikingly, in the apical hook, CMT orientation is opposite to the one observed in bending roots and stems (Baral *et al*., 2021). Thus, the enigmatic role of CMTs in bending needs to be addressed in order to bridge the scales from subcellular to organ growth asymmetry. By deepening our knowledge of these asymmetric growth mechanisms, we can gain a more comprehensive understanding of how diverse plant forms and adaptive strategies arise, ultimately enhancing our grasp of the principles underlying plant biology. This emphasizes the importance of growth asymmetry as a foundational concept in plant science, driving further discoveries in the field.

## Competing interests

None declared.

## Disclaimer

The New Phytologist Foundation remains neutral with regard to jurisdictional claims in maps and in any institutional affiliations.
